# Supplementary figures and images for: The R Language: An Engine for Bioinformatics and Data Science
Source: Life (Basel). 2022 Apr 27;12(5):648. doi: 10.3390/life12050648 (PMC9148156; doi:10.3390/life12050648)

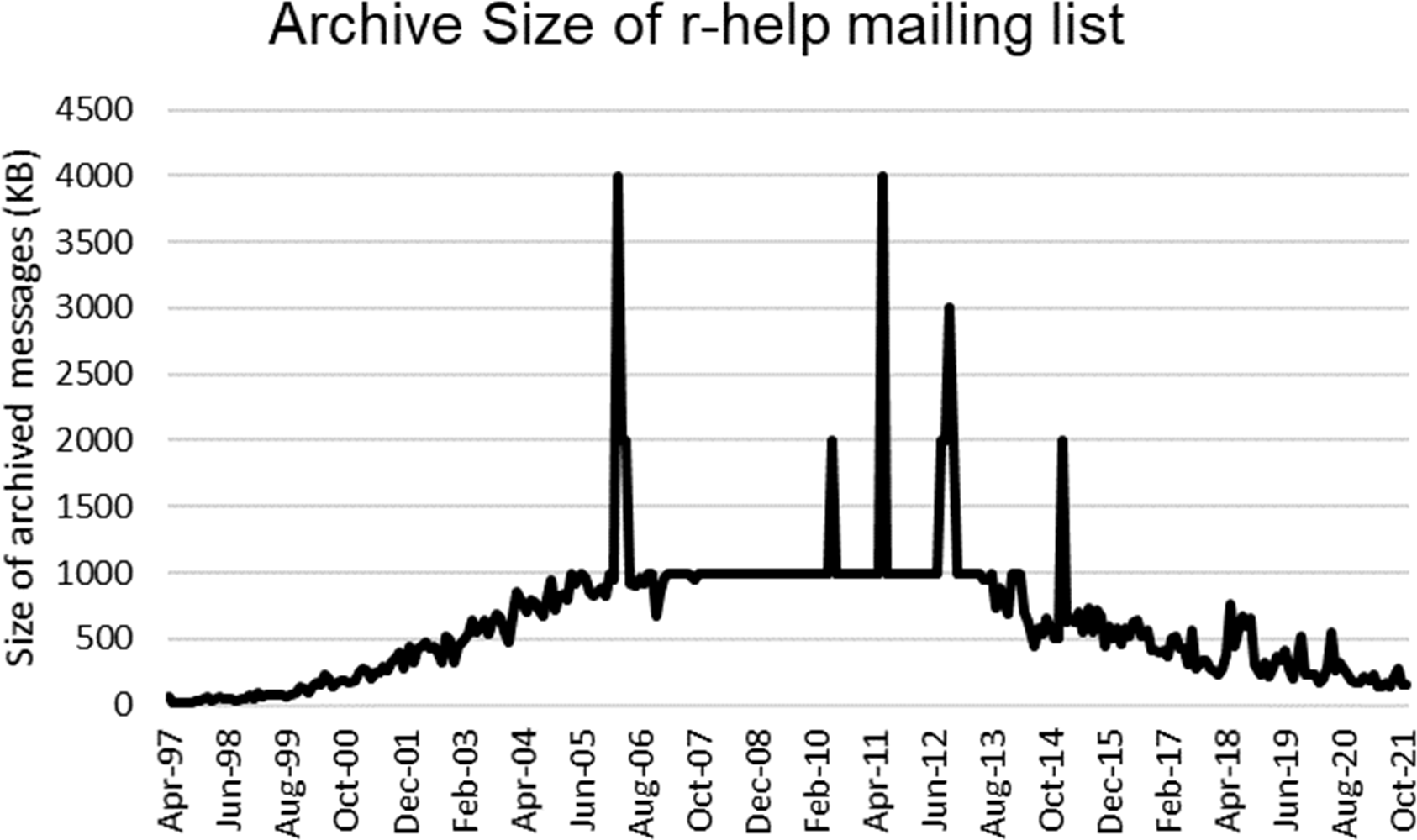

Supplement: Supplementary file 1 [file life-12-00648-s001.zip › SupplementaryFigureS1.TIF]

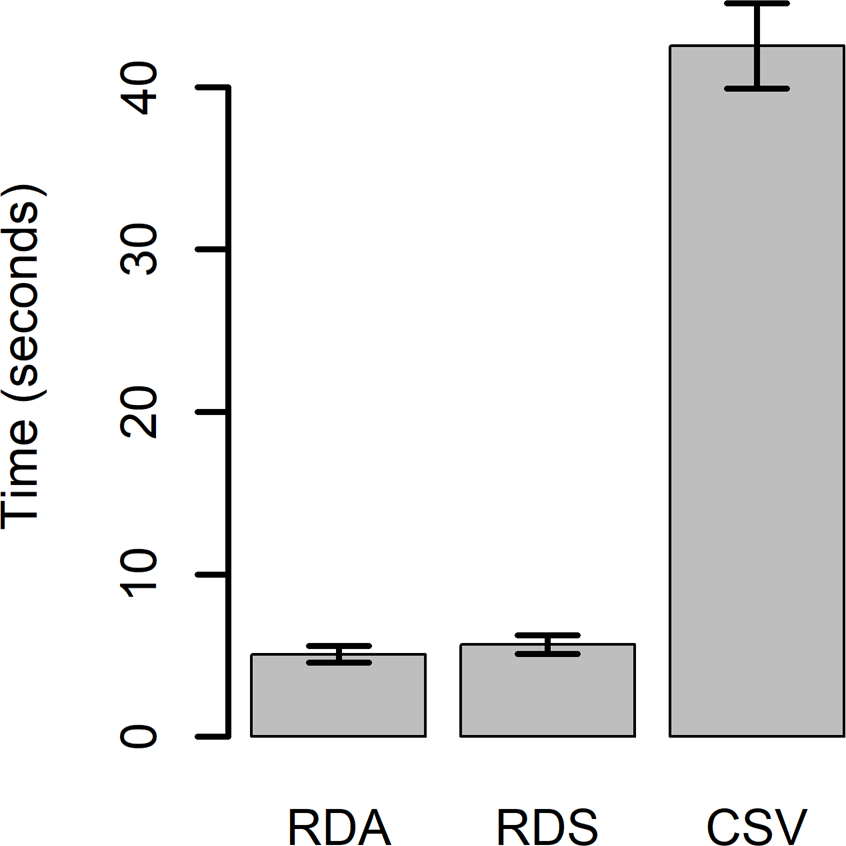

Supplement: Supplementary file 1 [file life-12-00648-s001.zip › SupplementaryFigureS2.TIF]
